# Multivariate Modelling Based on Isotopic, Elemental, and Fatty Acid Profiles to Distinguish the Backyard and Barn Eggs

**DOI:** 10.3390/foods13203240

**Published:** 2024-10-11

**Authors:** Gabriela Cristea, Florina-Dorina Covaciu, Ioana Feher, Romulus Puscas, Cezara Voica, Adriana Dehelean

**Affiliations:** National Institute for Research and Development of Isotopic and Molecular Technologies, 67-103 Donat Street, 400293 Cluj-Napoca, Romania; gabriela.cristea@itim-cj.ro (G.C.); florina.covaciu@itim-cj.ro (F.-D.C.); ioana.feher@itim-cj.ro (I.F.); romulus.puscas@itim-cj.ro (R.P.); cezara.voica@itim-cj.ro (C.V.)

**Keywords:** egg, stable isotopes, mineral concentration, heavy metals, fatty acids, chemometrics

## Abstract

The ability to trace the origin of eggs from backyard-raised hens is important due to their higher market value compared to barn-raised eggs. This study aimed to differentiate eggs from these two rearing systems using isotopic, elemental, and fatty acid profiles of egg yolks. A total of 90 egg yolk samples were analyzed, analytical results being followed by statistical tests (Student’s *t*-test) showing significant differences in δ^18^O, several elements (Mg, K, Sc, Mn, Fe, Ni, Cu, Zn, As, Cd, Ba, Pb), and fatty acids compositions (C23:0, C17:0, C18:0, C16:1n7, C18:1n9, C18:2n6, C20:1n7, C20:4n6, C20:5n3, C22:6n3), as well as in the ratios of SFA, PUFA, and UFA. The results indicated a nutritional advantage in backyard eggs due to their lower n-6 polyunsaturated fatty acid content and a more favorable n-6 to n-3 ratio, linked to differences in the hens’ diet and rearing systems. To classify the production system (backyard vs. barn), three pattern recognition methods were applied: linear discriminant analysis (LDA), k–nearest neighbor (k–NN), and multilayer perceptron artificial neural networks (MLP–ANN). LDA provided perfect initial separation, achieving 98.9% accuracy in cross-validation. k-NN yielded classification rates of 98.4% for the training set and 85.7% for the test set, while MLP–ANN achieved 100% accuracy in training and 92.3% in testing, with minor misclassification. These results demonstrate the effectiveness of fusion among isotopic, elemental, and fatty acid profiles in distinguishing backyard eggs from barn eggs and highlight the nutritional benefits of the backyard-rearing system.

## 1. Introduction

Worldwide egg production has a continuous growth over the last years, from 67 million tons in 2012 to 87 million tons in 2022 [1]. Proportionally, at the global level, per capita, egg consumption is rising as a result of several factors overlapping: the COVID-19 pandemic, the economic crisis, and African swine fever (which led to an increase in pork meat prices). Hen eggs are among the most nutritious foods on the planet, are not an expensive product, and contain high-quality protein [2,3,4,5].

The quality and composition of eggs vary significantly and are influenced by various factors, including the breed of the laying hens, their age, feeds (animal sources or plant), and production environment (industrial or free-range) [6]. There is research indicating that different hen farming methods significantly affect egg quality, nutritional content, and animal welfare. Organic systems generally provide superior benefits in these areas compared to conventional cage systems, aligning with growing consumer demand for ethically produced food products [7,8,9,10].

Distinguishing eggs coming from the backyard system versus the barn-growing system of hens is of great interest to consumers, regulators, and producers alike, as it provides valuable insights into production practices and potential differences in nutritional value. On the Romanian market, the price of an egg from a backyard chicken-rearing system is double compared with one from a barn-rearing system. Many consumers prefer to buy local products [11,12,13,14], backyard eggs being one of their choices, considering that these products have a better taste, a richer flavor, and higher nutritional value. They might be fresh, a day or two old, while barn eggs, bought from a supermarket, have a shelf-life of up to 28 days. The backyard eggs are sold directly through farmer’s markets, where conventional eggs, produced in a barn system, could be fraudulently sold as backyard eggs. Regarding the “backyard system”, eggs are produced by hens that are raised in a backyard or small-scale, home-based environment. The term “backyard eggs” suggests a more traditional way of raising hens. In barn egg farming, hens are housed in large sheds with a litter floor. There are slats for hens to roost and sleep on, and nest boxes are available for egg laying. In this context, there is an increasing need for proper analytical techniques to support consumer confidence in the value-added food market.

Some studies found that the cholesterol content in egg yolks varied depending on the farming method. For instance, eggs from hens supplemented with specific plant extracts showed a significant reduction in yolk cholesterol [15]. The fat content in yolks was assessed with free-range eggs, which sometimes exhibit lower fat levels but improved protein quality due to dietary differences [16]. Additionally, the nutritional composition of feeds used in different farming systems significantly impacted the egg’s nutrient profile. In the last decades, many important steps have been made in the traceability system and pattern recognition of the agri-food sector using different machine learning algorithms such as LDA, k–NN, MLP–ANN, etc. [17]. In the literature, there are a large number of papers reporting the benefits in the food industry brought by applying classical or advanced chemometric methods to analytical experimental data [18,19]. Among the strengths of using these machine learning methods in the food industry domain are quality control, food safety, product authentication, classification, prediction, and process optimization [20,21,22]. This approach might offer valuable information about data that otherwise could not be identified [23].

The goal of the present study was to develop chemometric models able to verify the eggs’ provenance system (backyard and barn) based on isotopic, elemental, and fatty acid profiles of egg yolks. The following steps were completed: (i) isotopic fingerprint (^13^C, ^2^H, ^18^O) assessment by Isotope Ratios Mass Spectrometry—IRMS; (ii) the determination of elemental content by Inductively Coupled Plasma Mass Spectrometry—ICP–MS; (iii) fatty acids profile quantification using Gas Chromatography Flame Ionization Detector—GC-FID; and (iv) the application of pattern recognition techniques, such LDA, k–NN and MLP-ANN. The combination of analytical techniques and multivariate modeling (LDA, k–NN, and ANN) to differentiate between backyard and commercial eggs represents a novel approach.

## 2. Materials and Methods

### 2.1. Samples Description

A total of 90 egg samples were collected from two distinct hens rearing systems: backyard (*n* = 45) and barn from conventional/industrial production facilities (*n* = 45), and investigated from the point of view of:

(i) Isotopic content;
(ii) Elemental concentrations and;
(iii) Fatty acids profiles.

The backyard egg samples were collected from trusted sources in different Romanian counties (e.g., Constanta, Alba, Cluj, Salaj, Mures, Suceava, Satu-Mare, Bistrita, and Sibiu). The hens raised in the backyard systems had outdoor space access to grinding in soil, pecking seeds, insects, worms, and grass, in addition to their feed given by their breeders (sunflower seeds, wheat, barley in the Southeastern part of the country, and corn flour, maize, wheat and barley in the central and the North part of Romania). Barn egg samples were bought from supermarkets and came from commercial Romanian egg producers. Barn-raised hens were housed in large, climate-controlled sheds without cages, allowing them to roam freely within the shed. Hens from conventional barn systems received a standardized commercial feed that was formulated to meet specific nutritional requirements.

Upon arrival at the laboratory, egg samples were visually inspected for any signs of damage, contamination, or irregularities. Each egg was individually identified and coded to maintain traceability throughout the analysis process. Every yolk was carefully separated from albumen and then homogenized separately to ensure uniformity. For further isotopic and elemental determinations, the water was extracted using cryogenic distillation under vacuum, utilizing liquid nitrogen [24]. At the end of this step, the obtained egg yolk was completely dry, without any water content.

In order to obtain the isotopic fingerprint, elemental content, and fatty acid composition, each egg yolk was prepared separately, conforming to a particular protocol. Hereinafter, the two types of hen growing systems will be referred to as BY (for backyard) and B (for barn).

### 2.2. Stable Isotopic Analysis

From egg yolk, both ^13^C isotopic fingerprints of bulk yolk and lipid-free yolk were determined. Then, the ^2^H and ^18^O isotopic signatures were recorded from the water extracted from the egg yolk.

During the sample preparation procedure, for ^13^C isotopic measurements, the analyte must be converted into CO_2_ gas. For this purpose, the dried yolk was homogenized by grinding with a mortar and pestle. After that, 5 mg of the sample was transformed into CO_2_ by dry combustion in oxygen excess, using an oven for 3 h at 550 °C. The resulting CO_2_ was separated from the other resulting gases by a cryogenic purification step and, afterward, measured using the IRMS technique.

Lipids were removed by rinsing yolk samples with a mixed solution of chloroform and methanol (1:2, *v*/*v*). This step of lipid extraction was completed due to the fact that the dry egg yolk contains more than 50% lipids, and this fraction, rich in fat content, presents depleted ^13^C values compared with proteins [25]. The obtained lipid-free yolk was kept in an oven for 48 h at 55 °C before the dry combustion stage for subsequent isotopic analysis.

The results, presented as isotopic compositions (fingerprints, signature of ^13^C, ^2^H, ^18^O), are reported in conventional δ notation versus international standards, in compliance with Equation (1) [26]:

δ_ref_ (^i^E/^j^E, sample) = [R (^i^E/^j^E, sample)/R (^i^E/^j^E, ref)] − 1 (1)

where ref is the international measurement standard, the sample represents the analyzed sample, and ^i^E/^j^E is the isotope ratio between heavier and lighter isotopes. The delta values are [24] multiplied by 1000 and expressed in units “per mil” (‰). The international standards are Vienna-Pee Dee Belemnite (V-PDB) for ^13^C/^12^C determinations and Vienna-Standard Mean Ocean Water (V-SMOW) for measurements of ^2^H/^1^H and ^18^O/^16^O.

To obtain the ^13^C fingerprint, bulk yolk, and delipidated yolk samples were analyzed using an isotope ratio mass spectrometer (Delta V Advantage, Thermo Scientific, Waltham, MA, USA) in line with a dual inlet system. All samples were analyzed in duplicate. Every day, one working standard was measured before the sample analysis started. This standard was calibrated using a certified reference material, NBS–22 oil, from IAEA (International Atomic Energy Agency), having δ^13^C_VPDB_ = −30.03‰. The uncertainty limit for ^13^C determinations from bulk and lipid-free yolk samples was ±0.2‰.

The equipment utilized for recording the isotopic fingerprints of water (^2^H and ^18^O), previously extracted from egg yolk, was a liquid-water isotope analyzer (DLT–100, Los Gatos Research, San Jose, CA, USA). The isotopic values were calibrated using a set containing five international working standards (WS) (WS 1, δ^18^O = −19.57‰ and δ^2^H = −154.1‰; WS 2, δ^18^O = −15.55‰ and δ^2^H = −117.0‰; WS 3, δ^18^O = −11.54‰ and δ^2^H = −79.0‰; WS 4, δ^18^O = −7.14‰ and δ^2^H = −43.6‰; WS 5, δ^18^O = −2.96‰ and δ^2^H = −9.8‰). All samples were measured in duplicate, the final result was obtained by the average, and each acquisition consisted of seven replicates. The uncertainty for δ^18^O was ±0.2‰ and ±1‰ for δ^2^H, respectively.

### 2.3. Elemental Profile Analysis

Elemental profile analysis was conducted to determine the concentrations of essential minerals and trace elements (Na, Mg, K, Ca, Sc, Ti, V, Cr, Mn, Fe, Co, Ni, Cu, Zn, As, Cd, Ba and Pb) in the egg yolk samples. The analysis was performed by inductively coupled plasma mass spectrometry (ICP-MS) using an ELAN DRC (e) mass spectrometer (Perkin Elmer SCIEX, Framingham, MA, USA), measuring each egg sample in duplicate. Prior to analysis, 0.1 g of dry samples (obtained by a procedure that used cryogenic distillation under vacuum) were digested using HNO_3_ 60% (*v*/*v*) (Sigma-Aldrich (Merck), St. Louis, MO, USA) and H_2_O_2_ 30% (*v*/*v*) (Chempur, Piekary Śląskie, Poland), according to instrumental parameters and settings reported previously [24]. The blank solution (4 mL of HNO_3_ (60% *w*/*w* %) and 1 mL of H_2_O_2_ (30% *w*/*w* %)) was used. After acid digestion, the resulting solutions were cooled to room temperature and then diluted to 50 mL with ultrapure water (resistivity 18 MΩ cm^−1^, Millipore, Bedford, MA, USA). The solutions were kept in the refrigerator until the moment of the elemental content analysis, using the selected analytical method. The spectrometer parameters were: nebulizer gas flow rates (0.92 L/min); auxiliary gas flow (1.2 L/min); plasma gas Flow (15 L/min); lens voltage (7.25 V); radiofrequency power (1100 W); CeO/Ce = 0.025; Ba^++^/Ba^+^ = 0.020. Multi-element calibration standard 2 (10 μg/mL) of Ce, Dy, Er, Eu, Gd, Ho, La, Lu, Nd, Pr, Sc, Sm, Tb, Th, Tm, Y, Yb, multi-element calibration standard 3 (10 μg/mL) of Ag, Al, As, Ba, Be, Bi, Ca, Cd, Co, Cr, Cs, Cu, Fe, Ga, In, K, Li, Mg, Mn, Na, Ni, Pb, Rb, Se, Sr, Tl, U, V, Zn were obtained from PerkinElmer Pure Plus, USA, and titanium standard for ICP (1000 mg/L) from Sigma-Aldrich, Switzerland were used for the standard stock solutions preparation, by dissolving the multi-element solutions with ultrapure water. For the calibration curve (for each element), the working solutions of a specific concentration and volume were prepared by diluting the stock solution.

### 2.4. Fatty Acids Profile Analysis

The fatty acid composition of egg yolk samples was examined using a slightly adjusted version of the previously established method [27]. Thus, prior to the analysis, approximately 0.1 g samples were weighed and placed in a 2 mL derivatization vial. To this vial, 0.5 mL of hydrolysis solution (0.5 M NaOH/CH_3_OH) and 10 μL of internal standard (methyl undecanoate) were added. Subsequently, the vial was heated in an oven at 100 °C for one hour. The extracted lipids were then derivatized into fatty acid methyl esters (FAMEs) to enhance their volatility and chromatographic separation. Once the vial was cooled to room temperature, 500 μL of a methylation solution (3M HCl/CH_3_OH) was introduced. The contents were mixed thoroughly using a vortex for 15 s and then placed back into the oven at 100 °C for 30 min. After cooling back to room temperature, 500 μL of n–hexane was added. The mixture was vortexed for 15 s and then centrifuged at 1000 rpm for 5 min. Finally, the upper organic layer was collected for analysis. Fatty acid analysis was conducted using gas chromatography with flame ionization detection (GC–FID) (Trace GC Ultra model by Thermo Electron Corporation, Milan, Italy) utilizing a DB–FATWAX column (30 m × 0.25 mm × 0.25 μm, manufactured by Agilent Technologies, Santa Clara, CA, USA). A 1 μL aliquot of the sample was injected with a split ratio of 10:1 at an injection and detection temperature of 250 °C. Helium gas of very high purity (99.999%) was used as the carrier gas at a constant flow rate of 1.8 mL/min. The temperature inside the oven was initially set at 50 °C for 2 min, then increased at 4 °C/min to 220 °C, where it was maintained for 20 min. The identification and quantification of fatty acids were carried out by comparing them with known fatty acid standards (Supelco^TM^ 37 Component FAME Mix) provided by Sigma-Aldrich Co. LLC, St. Louis, MO, USA. Fatty acid concentrations were expressed as percentages of total fatty acids, allowing comparisons of relative abundance between different samples.

### 2.5. Chemometric Analysis

Chemometric analysis was carried out using SPSS v.24 (IBM, New York, NY, USA) software. The working data matrix was obtained by a data fusion approach, meaning a combination of the experimental data obtained from the three employed analytical techniques (isotopic, elemental, and fatty acid profile results). The main goal of data fusion is to enhance the synergy between the fused techniques by using complementary inputs to finally obtain better classifications or prediction capabilities. This approach is performed for a better insight into an experimental data set and has emerged as a way to increase the reliability of classification or prediction compared to using a single analytical technique [28]. The objective was to identify patterns, relationships, and discriminative features within the data, enabling differentiation between egg yolks coming from backyard and barn production systems of hens.

Firstly, for testing the normality of data, Kolmogorov-Smirnov and Shapiro-Wilk tests were employed, and to identify significant differences between egg yolks (BY vs. B), the Student’s *t*-test was conducted. Any differences associated with *p*-values less than 0.05 were considered to be statistically significant.

Then, linear discriminant analysis (LDA) was applied, which is a widely employed supervised chemometric method used for classification purposes. This method creates a classification model containing linear combinations of original variables called discriminant functions (DFs). The performance of the LDA model is evaluated through the “leave-one-out cross-validation” method. In leave-one-out cross-validation (LOOCV), each sample is removed from the dataset, and the model is trained on the remaining data and then tested with the removed sample. This process is repeated for each sample, and the overall classification accuracy is assessed based on the model’s performance across all iterations. The LDA model performance was evaluated using metrics such as classification accuracy (The proportion of correctly classified samples out of the total number of samples), sensitivity, and specificity (provides insight into the model’s ability to correctly identify samples from each class (e.g., backyard vs. barn egg yolks)).

Besides the above-mentioned classification method, another widely employed statistical test is k–nearest neighbor (k–NN). k–NN is a supervised learning algorithm that can be used to handle both classification and prediction problems. As a prediction method, it relies on similarities of new samples with available data and places the sample to the closest group. k–NN follows the next stages: (1) estimates the distances between the sample test and each training point, (2) selects the k–nearest neighbors to the sample test, and (3) predicts the class or value of the new samples based on the majority class or the mean value of the neighbors, respectively. The accuracy of the k-NN prediction model is assessed, as for LDA, through the percent of correctly classified samples. In this case, the validation technique is the so-called “hold out method”, which divides the dataset into two sets of training and test subsets. The training dataset is used to train the model, and then test data is fitted into the trained model to make predictions. For k-NN, the success of the model heavily depends on the choice of k (the number of nearest neighbors). Due to its non-parametric nature, it is very suitable for real data sets, as in the case of the classification of egg yolks [22].

Apart from LDA, another supervised learning method is artificial neural networks (ANN), which is the best choice when complex demands need to be addressed. ANNs are computationally based mathematical tools inspired by the human nervous system, the neuron. ANNs constitute a simplified artificial replica of the human brain consisting of parallel processing neural elements similar to neurons in living beings. ANN is able to store large amounts of experimental information to be used for generalization with the aid of an appropriate prediction model [29]. The accuracy is expressed as the ratio between correct predictions and total predictions. Moreover, in the case of ANN, some more specific parameters are employed for performance evaluation. One is the sum of square error, which is a measure of the difference between the predicted and the actual value; the closer to one, the more accurate the results are. Other important parameters are represented by receiver operating characteristic (ROC), which is a graphical representation of the model’s sensitivity and specificity. Along with these parameters, the area under the curve (AUC) is another important tool used for performance evaluation. Commonly, it is being used to evaluate the effectiveness of ANN’s accuracy in prediction and classification, where an AUC of 1 represents a perfect test. ANN has many applications in many domains, such as agri-food science and technology, as well as biological, medical, economic, and meteorological purposes.

## 3. Results and Discussion

### 3.1. Isotopic Fingerprints of Egg Yolk

^13^C isotopic fingerprints of terrestrial plants have different values and functions of enzymatic carbon fixation: C3 and C4 pathways. Thus, C3 plants follow photosynthesis, during which the initial chemical product formed in the carboxylation reaction is a three-carbon molecule, phosphoglyceric acid. About 85% of plant species are part of the C3 category: cereals (wheat, rice, barley, oats), vegetables (potatoes, carrots, tomatoes), and fruits (apples, pears, grapes, etc.). Throughout C4 photosynthesis, a dicarboxylic acid with 4 carbon atoms is assembled. C4 plants include corn, sorghum, sugarcane, and millet. ^13^C isotopic values for C3 plants are ranging between −30 and −23‰ [30]. For C4 plants, the isotopic signature of ^13^C has a completely distinct variation interval, from −14 to −12‰. Thus, the different plants that make up the hen’s diet will contribute with their own isotopic signatures to the final ^13^C fingerprint of chicken meat and eggs, offering information about the C3 and C4 plants proportion added to the hen’s feeding regime.

For all samples, the isotopic composition of pairs bulk yolk–delipidated yolk had the same trend, with an average of 1.1‰ difference among values. The higher values were obtained for delipidated samples, similar to those presented in the literature [10], proving the importance of the dilapidation step during the sample preparation protocol. Taking into account this fact, discussion related to the ^13^C isotopic fingerprint of egg yolk will be referred to delipidated results.

**Figure 1:**
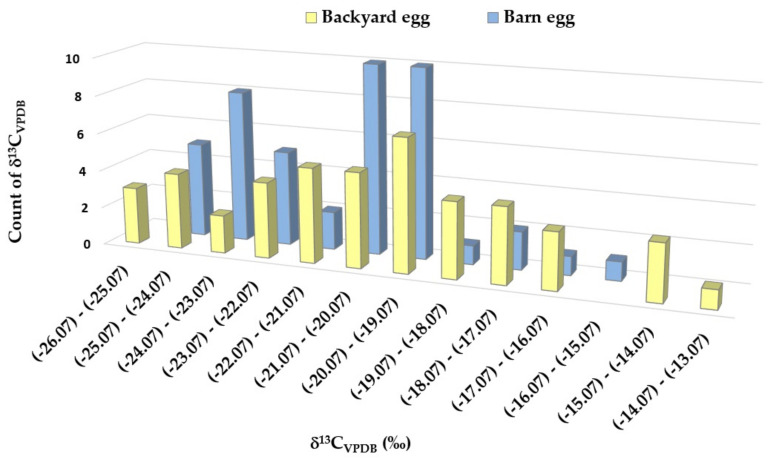
Chart of ^13^C isotopic values for egg yolk samples coming from two different rearing systems of hens.

δ^13^C values of lipid-free egg yolk varied between −26.1 and −14.0‰ for samples coming from the backyard rearing system and from −24.4 to −15.9‰ for samples originating from the barn system. To better emphasize the isotopic differences between these two different growing systems of hens from where the eggs are coming from, the isotopic values of ^13^C are presented in the chart below (Figure 1).

For the egg yolk samples coming from the backyard poultry system, a higher ^13^C isotopic signature compared to the samples originating from the industrial system was recorded. The isotopic composition of three samples from the BY set proves a C4 exclusive diet composed of maize (*Zea mays*) and sorghum (*Sorghum bicolor*). For nine samples from the BY group, a feeding regime consisting of C3 plants was identified, the corresponding ^13^C isotopic fingerprints having values below the threshold value of −22.5‰ proposed by United Kingdom researchers [31]. This critical value, suggested in a previous study [31], could allocate (>97.5%) chicken as non-corn fed if the diet contains no more than 23.3% corn. For the rest of the 33 BY samples, the ^13^C isotopic results confirm a mixed diet of C3 and C4 plants containing an important fraction of maize. Similar results were obtained in our preliminary study [24]. A rich corn-based diet represents an old tradition in Romania for yard swine and chicken growing systems [32,33]. These kinds of eggs are gaining more and more popularity among consumers and are greatly appreciated [34]. Chickens that are roaming the yard are pecking grass and bugs, getting more leafy greens and natural proteins than those raised in poultry houses that are likely getting fed pelleted feed [35,36]. Chickens that are raised in a small-scale setting are usually fed with food sources that are of a higher quality than those raised in the industrial system. Backyard hens spend their days outside, absorbing vitamin D, which leads to a much better-tasting egg [37]. Hens lay healthier when they are able to forage for themselves and are doing ordinary chicken activities, such as roosting and dust bathing [38].

Regarding egg yolk samples coming from the barn system, the isotopic signature of ^13^C proved a combined diet, formed of C3 and C4 plants, for 29 of the samples from the total set of 45, while for 16 samples, having results less than the critical value of −22.5‰, a C3 plant feeding regime of hens was identified. The mean value for barn set samples (yolk lipid-free) was δ^13^C_VPDB_ = −21.2‰. It fits the values reported by [10], ranging from −22.9 to −21.5‰ for delipidated yolk samples originating from the barn regime.

**Figure 2:**
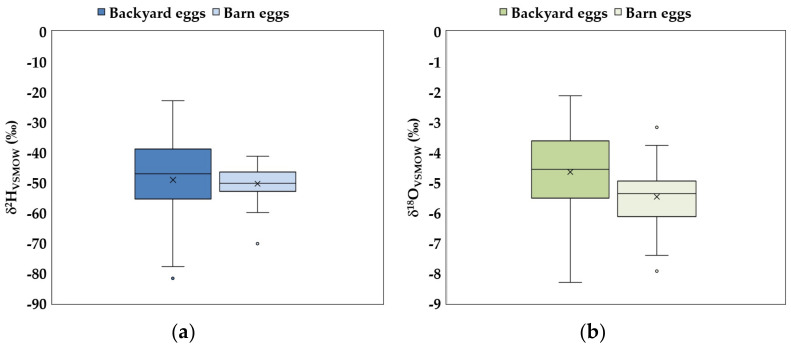
Box diagrams of (**a**) δ^2^H (a) and (**b**) δ^18^O for the egg yolk samples. The line across the boxes represents the median. Whiskers indicate the higher and lower values in the entire data range. The circles represent the outliers, and the cross symbol is the mean of the data.

With respect to isotopic compositions of extracted water from egg yolk, as can be observed in Figure 2, they ranged from −81.7 to −23.0‰ (BY) and from −70.3 to −41.3‰ (B) for ^2^H, while those of ^18^O varied between −8.3 and −2.1‰ (BY) and −7.9 to −3.2‰ (B).

As expected, the range of variation, both of δ^2^H and δ^18^O, is wider for BY samples compared to B samples. The explanation consists of the fact that backyard egg producers usually have their farms in the rural regions, covering many areas of the country. Precipitation that falls at a site will be transmitted to the drinking water from the respective location, which will be given to hens, and then the isotopic fingerprint of extracted water from egg yolk will be related to that territory [30,39,40]. Barn eggs come from industrial poultry farms; these farms are not so scattered throughout the country. Thus, the range of variation for water isotopes is narrower. This explanation is supported by [40], which differentiated the origin of bio-eggs from conventional eggs using stable isotopes. Its results highlighted the fact that the isotopic signatures of ^2^H and ^18^O can have very high values in ecological eggs; the geographical region is reflected in the isotopic values of the drinking water and then in the water of the egg. Boner [40] concluded that such high correlation factors of ^2^H and ^18^O could not be recorded for conventional farms, and, in the future, stable isotopes give the opportunity to differentiate between conventional and ecological eggs.

### 3.2. Elemental Profile of Egg Yolk

Considering that fresh eggs are among the most important foods in the daily diet, there is considerable interest in the elemental composition of hen eggs from the perspective of human health [41]. In the current study, the elements analyzed in egg yolk samples include Na, Mg, K, Ca, Sc, Ti, V, Cr, Mn, Fe, Co, Ni, Cu, Zn, As, Cd, Ba, Pb (see Table 1). The relative standard deviation (RSD) of measurements was below 10% for most elements in the studied egg yolk samples. Some of these, such as Ca, Mg, K, Na, Fe, Zn, Cu, Cr, and Mn, play vital roles in various physiological processes [42,43,44] and are important indicators of egg quality and nutritional value. The average content of macro minerals (Na, Mg, K, and Ca) and micro essential elements (Fe, Zn, Cu, Mn, and Cr) obtained in this study showed the following order: Ca > K>Na > Mg > Fe > Zn > Cu > Cr > Mn (for samples coming from BY system) and Ca > K>Na > Mg > Fe > Zn > Cr > Cu > Mn (for B system). BY egg yolks have a higher content of Na, Mg, K, Mn, Fe, Cu, and Zn compared to samples coming from the B system. The mean trace elements concentrations (Sc, Ti, V, Co, Ni, As, Cd, Ba and Pb), in mg/kg fresh weight, in yolk samples coming from BY hens’ system were 0.20, 1.04, 0.20, 0.02, 0.50, 0.04, 0.04, 8.84 and 0.09, and 0.16, 0.84, 0.16, 0.02, 0.23, 0.02, 0.02, 1.36 and 0.02 for egg yolks from the B system, respectively. It is noteworthy that yolk samples from the B system have the smallest content of toxic elements (As, Cd, Pb). The European legislation [45] has set permissible limits on Pb, Cd, and Hg in certain foods, but not for eggs.

The results obtained by Student’s *t*-test showed significant differences (*p* < 0.05) among the samples coming from nominated production systems regarding the concentrations of Mg, K, Sc, Mn, Fe, Ni, Cu, Zn, As, Cd, Ba, and Pb measured in egg yolks. In our study, the concentrations of Na, Mg, K, Sc, Mn, Fe, Ni, Cu, Zn, As, Cd, Ba, and Pb in yolk samples from the BY system were higher than those recorded for samples from B system, as it can be observed in Table 1. These differences may be attributed to variations in the composition of the hens’ diets, including the types and amounts of mineral supplements provided. The courtyard hens are exposed to more variable environmental conditions (free access to soil, availability of grass, or different types of ingested feed), which could contribute to an additional daily intake of the essential and non-essential elements compared to the stable conditions in industrial farms. Table 2 shows the content comparison of some elements studied in the present study with data provided by different authors [24,46,47,48,49].

#### 3.2.1. Major Elements (Ca, K, Mg, Na)

The concentrations of Ca in the present study (1309.45 mg/kg in BY system, 1316.07 mg/kg in B system) align well with recent findings from 2023 [24], which reported similar values in mg/kg (1350.70-BY system, 1364.54-B system). However, older data from [49] (1999) suggested slightly higher calcium levels in different farms (1300–1400 mg/kg), potentially due to differences in feeding or environmental conditions. K values (in mg/kg) in the present study (1281.37-BY system, 962.20-B system) also closely match those reported in 2023 [24] (1292.95-BY, 1028.51-B, in mg/kg), but they fall within the range of earlier results (1200–1400 mg/kg) from [49] (1999). The consistency across studies suggests that farming systems influence potassium levels, with higher levels in backyard-produced eggs. For Mg (in mg/kg), our results (181.09-BY, 147.80-B) are consistent with [24] (180.08-BY, 164.24-B, in mg/kg) and higher than older reports [48] (2005), [49] (1999) where values ranged from 150 to 160 mg/kg. This slight increase may be attributed to diet variations or improved farming practices over time. Na concentrations (856.71-BY, 770.97-B, in mg/kg) were also similar to [24] (887.12-BY, 830.47-B in mg/kg), while older studies [49] reported significantly lower sodium levels (500 mg/kg). These differences could be due to advancements in feed formulations.

#### 3.2.2. Trace Elements (Cr, Cu, Fe, Mn, Ni, Pb, V, Zn)

Cr levels in the present study (1.92-BY, 1.93-B in mg/kg) are lower than those reported by [24] (3.28-BY, 3.16-B, mg/kg). However, much older studies like [47] (2009) reported significantly lower values (0.066–0.090 mg/kg), which could reflect a trend of increasing Cr levels in more recent years, possibly linked to environmental or dietary factors. Cu concentrations (2.12-BY, 1.33-B, mg/kg) in this study are slightly lower than in [24] (2.40-BY, 2.35-B, mg/kg) and older results from [46] (2013) (10.5 mg/kg), indicating possible variations in feed composition or farming practices. Fe concentrations (123.27-BY, 92.81-B in mg/kg) in the present study are somewhat higher than in [24] (114.27-BY, 94.06-B, in mg/kg) and significantly higher than values (61–70 mg/kg) [49], suggesting increased iron content in modern egg production. For Mn, the current study reports levels (1.54-BY, 1.03-B in mg/kg) comparable to [24] (1.34-BY, 1.21-B in mg/kg) but significantly lower than earlier findings [46] (6.8 mg/kg). Variations may be due to differences in diet and farm conditions. Ni concentrations in the present study (0.50-BY, 0.23-B in mg/kg) were lower than in older studies [47] (1.7–3.1 in mg/kg), which could reflect reduced environmental contamination over time. For Pb, the current study reports trace levels (0.09-BY, 0.02-B in mg/kg), similar to [24] (0.06-BY, 0.03-B in mg/kg), reflecting low contamination, likely due to regulatory controls on lead exposure in food production. V levels in our study (0.20-BY, 0.16-B, mg/kg) were notably higher than the trace values (0.012–0.013 mg/kg) reported in [48] (2005), indicating potential environmental exposure differences between regions. Lastly, Zn concentrations (34.34-BY, 28.68-B in mg/kg) were comparable to [24] (30.6-BY, 28.00-B in mg/kg) but slightly lower than older studies [46] (35.6–42.2 mg/kg) and [49] (39–40 mg/kg), possibly due to differences in feed supplementation practices across different time periods.

Overall, the elemental concentrations reported in the present study generally align with those from recent literature, although there are some variations that could be attributed to differences in farming systems, environmental conditions, and advancements in feed formulations. In particular, trace elements like Cr, Ni, and V show notable variations, which may be due to evolving environmental and agricultural practices. These comparisons highlight the dynamic nature of elemental composition in egg yolks, influenced by both production methods and temporal changes.

### 3.3. Fatty Acid Profile of Egg Yolk

The fatty acid composition of eggs is considered a crucial quality factor for consumers, leading to initiatives aimed at improving the fatty acid profile of eggs through dietary changes [50]. Our analysis revealed a significant variation in the fatty acid profiles of egg yolks from backyard systems compared to those from barn systems. The fatty acid profile analysis of egg yolks is presented in Table 3. Regarding the fatty acid composition of egg yolk, the proportions of stearic acid (C18:0) and total saturated fatty acids (SFAs) in the lipid of egg yolks from the backyard were higher than that in barn eggs. By comparison, backyard yolk eggs exhibited lower palmitic acid (C16:0) content than barn yolk eggs. The fatty acid composition of the yolk samples was aligned with findings from previous research [51].

Specifically, while the egg yolks from backyard systems had a higher concentration of n-3 polyunsaturated fatty acids (n-3 PUFAs), particularly C18:3n3, C20:5n3, and C22:6n3, eggs from barn systems exhibited a higher ratio of n-6 to n-3 PUFAs, raising concerns due to the already excessive levels of n-6 PUFAs in the modern diet. This distinction emphasizes the influence of a hen’s diet and living conditions on egg nutrition, as backyard hens typically have access to a more varied and natural diet. Barn egg production often prioritizes efficiency and consistency, which can lead to differences in nutrient content compared to eggs from the backyard or small-scale operations where hens have more diverse diets [50]. In addition, total monounsaturated fatty acids (MUFAs) were higher in backyard eggs, while polyunsaturated fatty acids (PUFAs) were more prevalent in barn eggs. This distinction highlights the nutritional benefits of backyard eggs, aligning with recommendations for heart health and chronic disease prevention.

**Figure 3:**
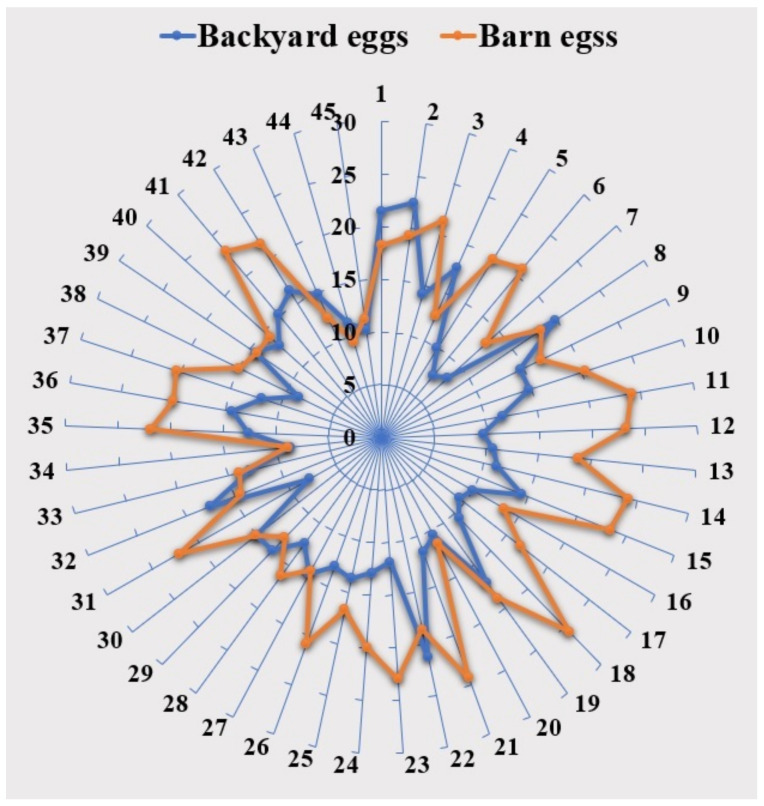
Radar chart comparison of n-6 PUFA concentrations in egg yolks from backyard (blue line) and barn (orange line) growing systems.

Backyard eggs contained 29.25% C18:1n9, 13.29% C18:2n6, 0.12% C18:3n6, 0.44% C18:3n3, 2.66% C22:6n3, 50.44% SFAs, 31.81% MUFAs, 17.75% PUFAs and 0.35 PUFAs/SFAs. The SFAs and MUFAs content in barn eggs ranged from 37.53% to 59.10% and from 20.89% to 42.34%, respectively. However, total n-3 PUFAs were lower and n-6 PUFAs higher by approximately 5% than in backyard eggs. The content of C18:2n6 and total n-6 PUFA is higher in barn eggs compared to backyard eggs, as can be seen in the radar chart (Figure 3). Finally, the total PUFA content and the PUFA/SFA ratio in the lipids of barn egg yolks were higher than in backyard eggs. The n-6/n-3 PUFA ratio in the backyard egg yolk was approximately two percent lower than in the barn egg yolk (3.49 and 4.65, respectively). It should be noted that, despite the absence of significant differences, backyard eggs were characterized by the numerically lowest n-6 PUFA content and the most desirable n-6/n-3 PUFA ratio. These differences may be attributed to variations in the hens’ diets, taking into account that backyard hens have access to a wider range of natural sources, such as insects and greens.

Among the polyunsaturated fatty acids (PUFA) in egg yolk, those that provide the most health benefits include linoleic acid (LA (C18:2n6)), essential for the synthesis of other long–chain fatty acids), linolenic acid (ALA (C18:3n3)), eicosapentaenoic acid (EPA (C20:5n3)) and docosahexaenoic (DHA (C22:6n3)) [52]. Most of these fatty acids are part of the n-3 PUFA family, which is known to support cardiovascular health, help brain development and cognitive functions, and lower the risks associated with cancer, autoimmune disorders, and diabetes [53]. Since the human body does not produce PUFA, they must be ingested through our diet. The fatty acid composition of egg yolks can vary based on factors such as the diet of the hens and whether the eggs are from backyard or barn sources. In general, eggs from pasture-raised or backyard hens tend to have higher levels of beneficial omega-3 fatty acids than eggs from hens raised in conventional operations, where the diet may be predominantly composed of grains [54]. Westernized diets are characterized by high consumption of n-6 PUFA, which raises the n-6/n-3 PUFA ratio in a range of 10:1 to 20:1, which increases the risk of developing inflammatory diseases like obesity [55,56]. Following the World Health Organization (WHO) guidelines for fat consumption, an optimal dietary ratio of n-6 to n-3 PUFA is recommended to be 4:1 [57].

Statistical analysis (Student’s *t*-test) indicated that contents of C23:0, C17:0, C18:0, C16:1n7, C18:1n9, C18:2n6, C20:1n7, C20:4n6, C20:5n3, C22:6n3, SFA, PUFA and UFA were significantly different between backyard and barn eggs (*p* < 0.05), as it can be observed in Table 3. The analytical results obtained by the three used techniques (IRMS, ICP–MS, and GC–FID) highlighted that backyard eggs presented higher concentrations of δ^13^C, Na, Mg, K, Sc, Mn, Fe, Cu, Zn, total monounsaturated fatty acids (MUFAs), and of n-3 polyunsaturated fatty acids (n-3 PUFAs) compared to barn eggs, suggesting the nutritional benefits of backyard eggs, according to guidelines for maintaining heart health and preventing chronic diseases.

### 3.4. Chemometric Models Development Based on Isotopic, Elemental, and Fatty Acid Profiles of Egg Yolks

**Figure 4:**
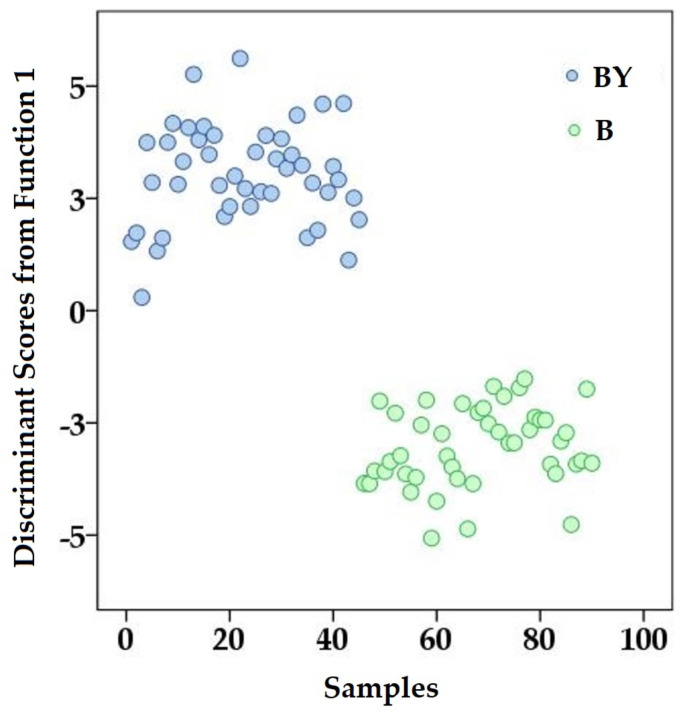
Discriminant scores for egg yolk samples provided by the LDA model.

To fulfill the aim of the present study, three supervised pattern recognition techniques, LDA, k–NN, and multilayer perceptron artificial neural network (MLP-ANN), were used. The first classification model was developed based on LDA in order to reveal some features that differentiate the two groups of egg yolks (backyard and barn). In this light, the specific markers were selected only from fatty acids and from multi-elemental content, as follows: C15:0, C17:0, C17:1, C18:0, C18:2n6, C18:3n3, C20:1n7, C23:0, and Fe, Ca, Co, and V. The standardized discriminant function coefficients were: C15:0 (−1.006), C17:0 (1.473), C17:1 (0.298), C18:0 (0.481), C18:2n6 (−1.359), C18:3n3 (0.604), C20:1n7 (−0.727), C23:0 (0.768) and Fe (0.737), Ca (−0.530), Co (0.3247) and V (−0.273). The model provided only one single discriminant function (DF1), which explained the entire variance of the dataset. Using the model obtained through LDA, an initial separation of 100% was obtained, while for cross-validation, the value was 98.9% (Figure 4).

Besides the high value obtained for accuracy, other performance parameters, such as sensitivity (97.8%) and specificity (100%), demonstrated the adequacy of this model for the prediction of egg yolk.

Among the machine learning algorithms, the k–NN is the simplest one, thus being very suitable for prediction purposes. In this case, this method was applied for the identification of egg yolk sources (backyard or barn-grown hens). As for the other classification method exposed above, the newly created variable contained a specific code for each category of egg yolk (1 for backyard-grown hens and 2 for barn-grown hens). The following specific parameters were set: the target variable was the newly created variable, and all measured experimental parameters were selected as features. The number of neighbors was automatically selected (between 3 and 5), while the computation distance was Euclidian measure. Also, the features were weighted by importance when the distances were computed. The maximum number of features was set to 10. The sample set was randomly split into a 70% training set and a 30% testing set.

The results were conducted to very good correct prediction percentages, 98.4% for the training set and 85.7% for the testing set. For the first stage, only one sample was misclassified from the backyard group to the barn group, while in the second phase, four samples were put to the incorrect group (Table 4).

**Figure 5:**
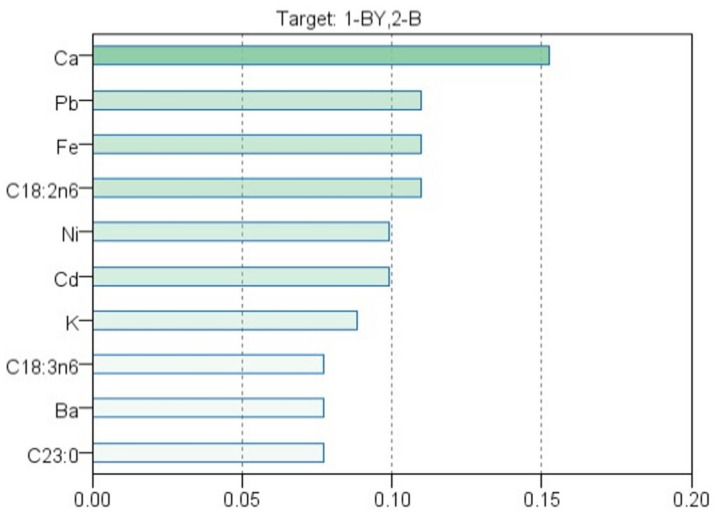
The importance of variables was identified using the k–NN algorithm.

**Figure 6:**
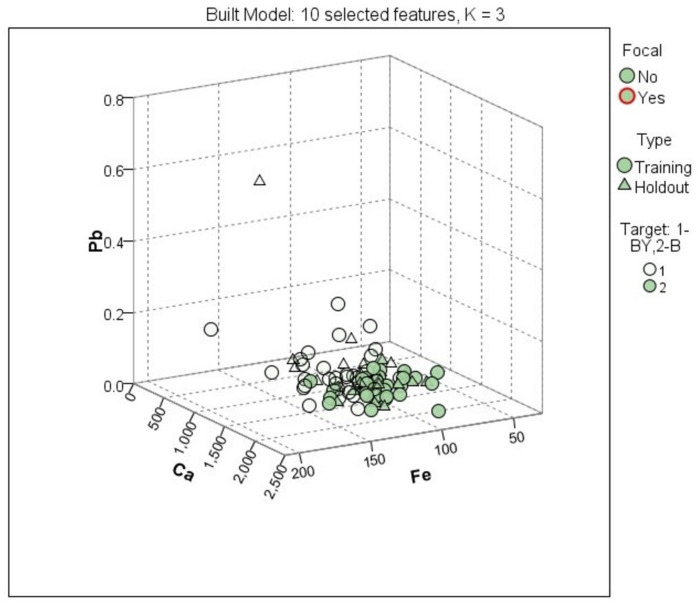
Samples distribution using the first three selected features.

In Figure 5 and Figure 6, the most significant variables among all the analyzed features, such as Ca, Pb, and Fe, can be observed. Additionally, fatty acids C18:2n6 and C18:3n6 also made a notable contribution. 

The sample distribution is crucial in chemometric analysis as it helps visualize how well the selected features separate or cluster different groups-in this case, eggs from backyard and barn systems. The distribution of samples along key variables (like Ca, Pb, and Fe) allows us to assess the effectiveness of the selected features in distinguishing between different categories. As shown in Figure 5 (or the 3D scatter plot), the clustering of the samples indicates the degree of separation between backyard (BY) and barn (B) eggs. Proper sample distribution ensures that classification models such as linear discriminant analysis (LDA), k–nearest neighbor (k–NN), and multilayer perceptron artificial neural networks (MLP–ANN) can effectively identify patterns that differentiate these two systems. Clarifying this link between sample distribution and model accuracy will further strengthen the analysis and underline the significance of the chosen features.

Some of the obtained markers, such as Ca, Fe, and C18:2n6, are also confirmed by the Student’s *t*-test and LDA, but some of them are new, a fact that led us to the conclusion that even both LDA and k–NN are classification methods, it could be used together, having a complementary character.

In order to improve the prediction rate of egg yolks, a non-linear pattern recognition technique, such as MLP–ANN, was applied. In this type of network, the neuron layers (input, hidden, and output) are connected by a feedforward connection. In the case herein, the input layer consists of 44 units corresponding to each experimental analyzed parameter. All variables were standardized before any other step. The partition of the data set was randomly assigned between training (70%) and testing (30%) steps. The MLP–ANN architecture was automatically selected to have a number of units between 1 and 50 in the hidden layers. The type of training was in the batch mode, with a scale conjugate gradient as an optimization algorithm. Hyperbolic tangent and Softmax functions were used in the hidden and output layers, respectively.

The egg yolk classification after running the MLP-ANN is presented in Table 5. The overall percentage for the training set was 100%, while for the testing step, it decreased to 92.3%, with two samples being misclassified. Moreover, other ROC representations reveal two “high curves” (one curve for each category), which means that the prediction model is very close to a perfect one. At the same time, the value for AUC is 0.996.

As the previous analysis revealed, the strongest predictors were represented by C23:0, C17:O, followed by C18:2n6. Chemometric processing enabled the development of robust and reliable models for differentiating eggs from backyard versus barn hens based on their isotopic data, elemental profiles, and fatty acid compositions.

## 4. Conclusions

Studying egg yolk samples, by the corroboration of three important analytical techniques (IRMS, ICP–MS, and GC–FID) with chemometric analysis (LDA, k–NN, and ANN), yielded valuable insights into the comprehensive evaluation of the growing system from where the hens are coming from (backyard and barn).

^13^C isotopic fingerprint of investigated samples presented higher values for BY samples due to a rich-corn-based diet, this diet type representing an old tradition for animal feeding regimes in small-scale farms in Romania. In regard to eggs originating from the barn system, the ^13^C recorded signature demonstrated a combined diet, formed of C3 and C4 plants, for 29 of the samples from the total set of 45, while for 16 samples, an exclusive C3 plants feeding regime of hens was identified.

Elemental analysis revealed variations in the concentrations of essential minerals and trace elements among investigated egg yolk types. The concentrations of Na, Mg, K, Sc, Mn, Fe, Ni, Cu, Zn, As, Cd, Ba, and Pb in yolk samples from the BY system were higher than those recorded for B samples, potentially attributable to the variable environmental conditions (free access to soil, availability of grass or different types of ingested feed), compared to the stable conditions in the industrial farms.

The fatty acid profile highlights the nutritional superiority of backyard eggs over barn eggs, particularly in their lower n-6 polyunsaturated fatty acid content and more favorable n-6 to n-3 PUFA ratio, emphasizing the critical role of the hens’ diet and living conditions.

LDA revealed a percentage of 100% in initial classification, while a percentage of 98.9% in the cross-validation procedure was reached based on the following significant parameters: C23:0, C18:2n6, C17:0, C20:1n7, C15:0, C18:0, C18:3n3, C17:1, Fe, Ca, Co and V. From k–NN analysis, the overall classification rate was 98.4% for training set and 85.7% for testing set. The most important features were Ca, Pb, Fe, Ni, Cd, K, Ba, C18:2n6, and C18:3n6. After running the MLP–ANN, the overall percentage for the training set was 100%, while for the testing step, it decreased to 92.3%, with two samples being misclassified. The strongest predictors were represented by C23:0, C17:0, followed by C18:2n6.

This study highlights the potential of combining analytical and chemometric techniques to perform comprehensive food quality assessments. In the future, the development of standardized protocols based on these methodologies could be useful to regulatory agencies, producers, and consumers, ensuring the integrity and transparency of the food supply chain.

## Figures and Tables

**Table 1:** The minimum (min), maximum (max) values (expressed in mg/kg fresh weight), relative standard deviation (RSD), and level of significance (*p*-value) of investigated elements in egg yolk samples.

Elements	BY				B				*p*-Value
	Min	RSD (%)	Max	RSD (%)	Min	RSD (%)	Max	RSD (%)	
Na	401.78	0.25	1501.64	0.12	436.44	0.25	1273.28	0.23	0.052
Mg	48.82	0.72	356.72	0.30	64.00	0.44	233.73	0.40	**0.000**
K	508.81	0.30	1941.69	0.18	486.56	0.49	1433.13	0.87	**0.000**
Ca	284.07	0.76	1851.98	0.23	857.18	0.22	2158.40	0.16	0.916
Sc	0.11	11.45	0.31	4.06	0.07	10.98	0.35	3.60	**0.000**
Ti	0.35	3.60	4.39	1.62	0.22	5.73	3.71	1.40	0.202
V	0.05	12.60	0.48	3.60	0.03	13.67	1.12	1.55	0.225
Cr	0.73	2.37	4.22	1.74	0.42	3.00	6.18	1.24	0.965
Mn	0.49	2.66	4.71	1.14	0.44	2.90	1.85	1.99	**0.000**
Fe	70.61	0.10	200.98	0.004	42.21	0.17	155	0.91	**0.000**
Co	0.01	14.00	0.08	13.63	0.002	7.05	0.07	2.02	0.064
Ni	0.02	7.07	4.41	1.18	0.01	3.4	0.67	2.58	**0.016**
Cu	0.03	4.71	7.39	0.96	0.02	5.48	2.67	1.57	**0.001**
Zn	21.38	0.67	105.73	0.78	18.82	1.05	69.02	0.39	**0.011**
As	0.005	2.80	0.08	1.77	0.001	14.00	0.08	1.77	**0.009**
Cd	0.01	1.29	0.43	2.97	0.003	4.24	0.05	2.61	**0.014**
Ba	1.62	1.06	67.09	0.16	0.33	3.98	14.77	0.23	**0.000**
Pb	0.01	12.29	0.71	2.42	0.002	2.78	0.10	4.23	**0.000**

BY-backyard system, B-barn system. The values marked with bold are considered statistically significant.

**Table 2:** The comparison of some element’s concentration (mg/kg fresh weight) was investigated in the present study with other authors.

Element	Present Study, 2024	[24], 2023	[46], 2013	[47], 2009	[48], 2005	[49], 1999
			Different Hen Farms			
Ca	1309.45-BY 1316.07-B	1350.70-BY 1364.54-B	-	-	1300	1400
K	1281.37-BY 962.20-B	1292.95-BY 1028.51-B	-	-	1200	1400
Mg	181.09-BY 147.80-B	180.08-BY 164.24-B	-	-	150	160
Na	856.71-BY 770.97-B	887.12-BY 830.47-B	-	-	500	500
Cr	1.92-BY 1.93–B	3.28-BY 3.16-B	-	0.066, 0.082 0.090	-	-
Cu	2.12-BY 1.33-B	2.40-BY 2.35-B	10.5 2.7	1.357	-	3.5
Fe	123.27-BY 92.81-B	114.27-BY 94.06-B	-	-	61	70
Mn	1.54-BY 1.03-B	1.34-BY 1.21-B	1.9 6.8	0.836, 0.797 0.705	-	0.5-2
Ni	0.50-BY 0.23-B	-	1.7 3.1	0.063, 0.058 0.059	-	-
Pb	0.09-BY 0.02-B	0.06-BY 0.03-B	-	-	-	-
V	0.20-BY 0.16-B	-	-	0.012, 0.013 0.012	-	-
Zn	34.34-BY 28.68-B	30.6-BY 28.00-B	35.6 42.2	20.68, 18.22 21.19	39	40

BY-backyard system, B-barn system.

**Table 3:** Fatty acid composition in yolks from backyard and barn eggs (expressed in % of total fatty acids) and level of significance (*p*-value).

Fatty Acid	BY	B	*p*-Value
	Concentration (%, Mean ± SD)		
C12:0	**0.01 ± 0.04**	0.01 ± 0.01	0.324
C14:0	0.37 ± 0.10	0.35 ± 0.06	0.412
C15:0	0.07 ± 0.03	0.08 ± 0.02	0.393
C16:0	27.36 ± 2.26	28.42 ± 2.92	0.058
C17:0	0.21 ± 0.08	0.14 ± 0.04	**0.000**
C18:0	22.13 ± 5.01	19.04 ± 4.73	**0.003**
C23:0	0.29 ± 0.12	0.17 ± 0.05	**0.000**
SFA	50.44 ± 5.23	48.20 ± 5.13	**0.043**
C14:1	0.07 ± 0.04	0.08 ± 0.04	0.111
C16:1n7	2.06 ± 0.84	2.70 ± 1.28	**0.005**
C17:1	0.27 ± 0.29	0.22 ± 0.09	0.292
C18:1n9	29.25 ± 4.93	26.75 ± 4.81	**0.017**
C20:1n7	0.16 ± 0.08	0.20 ± 0.08	**0.038**
MUFA	31.81 ± 5.19	29.95 ± 5.57	0.105
C18:2n6	13.29 ± 3.60	17.92 ± 4.39	**0.000**
C18:3n6	0.12 ± 0.05	0.11 ± 0.03	0.351
C18:3n3	0.44 ± 0.30	0.49 ± 0.33	0.367
C20:2n6	0.20 ± 0.08	0.21 ± 0.05	0.585
C20:4n6	0.19 ± 0.08	0.14 ± 0.04	**0.001**
C22:6n3	2.64 ± 0.64	2.28 ± 0.35	**0.001**
C20:5n3	0.87 ± 0.45	0.70 ± 0.29	**0.018**
PUFA	17.75 ± 3.63	21.85 ± 4.85	**0.000**
UFA	49.56 ± 5.23	51.80 ± 5.13	**0.043**
MUFA/SFA	0.63 ± 0.39	0.62 ± 0.49	0.798
PUFA/SFA	0.35 ± 0.19	0.45 ± 0.25	**0.000**
UFA/SFA	0.98 ± 0.87	1.07 ± 0.92	0.055
n-6 PUFA	13.80 ± 3.81	18.38 ± 4.51	**0.000**
n-3 PUFA	3.95 ± 1.39	3.46 ± 0.97	**0.003**
n-6/n-3 PUFA	3.49 ± 2.73	5.31 ± 4.65	**0.000**

BY-backyard system, B-barn system. The values marked with bold are considered statistically significant.

**Table 4:** Predictions were obtained using the k–NN algorithm for 90 egg yolk samples.

Partition		Predicted		
		BY	B	Percent Correct
Training	BY	32	1	96.97%
	B	0	29	100%
	Overall percent	51.62%	48.39%	98.39%
Holdout	BY	9	3	75.00%
	B	1	15	93.75%
	Overall percent	35.72%	64.29%	85.72%

BY-backyard system, B-barn system.

**Table 5:** MLP–ANN prediction for 90 egg yolk samples.

		Predicted		
Sample	Observed	BY	B	Percent Correct
Training	BY	32	1	100.0%
	B	0	29	100.0%
	Overall percent	54.7%	45.3%	100.0%
Testing	BY	9	1	90.0%
	B	1	15	93.8%
	Overall percent	38.5%	61.5%	92.3%

## Data Availability

The original contributions presented in the study are included in the article, further inquiries can be directed to the corresponding author.
